# ImmunoFISH: Simultaneous Visualisation of Proteins and DNA Sequences Gives Insight Into Meiotic Processes in Nuclei of Grasses

**DOI:** 10.3389/fpls.2018.01193

**Published:** 2018-08-14

**Authors:** Adél Sepsi, Attila Fábián, Katalin Jäger, J. S. Heslop-Harrison, Trude Schwarzacher

**Affiliations:** ^1^Department of Plant Cell Biology, Centre for Agricultural Research, Hungarian Academy of Sciences, Martonvásár, Hungary; ^2^Department of Genetics and Genome Biology, University of Leicester, Leicester, United Kingdom

**Keywords:** meiosis, cytology, chromatin dynamics, immunolabelling, ImmunoFISH, chromatin morphology

## Abstract

ImmunoFISH is a method combining immunolabelling (IL) with fluorescent *in situ* hybridisation (FISH) to simultaneously detect the nuclear distribution of proteins and specific DNA sequences within chromosomes. This approach is particularly important when analysing meiotic cell division where morphogenesis of individual proteins follows stage-specific changes and is accompanied by a noticeable chromatin dynamism. The method presented here is simple and provides reliable results of high quality signal, low background staining and can be completed within 2 days following preparation. Conventional widefield epifluorescent or laser scanning microscopy can be used for high resolution and three-dimensional analysis. Fixation and preparation techniques were optimised to best preserve nuclear morphology and protein epitopes without the need for any antigen retrieval. Preparation of plant material involved short cross-linking fixation of meiotic tissues with paraformaldehyde (PFA) followed by enzyme digestion and slide-mounting. In order to avoid rapid sample degradation typical of shortly fixed plant materials, and to be able to perform IL later, slides were snap-frozen and stored at -80°C. Ultra-freezing produced a remarkable degree of structural preservation for up to 12 months, whereby sample quality was similar to that of fresh material. Harsh chemicals and sample dehydration were avoided throughout the procedure and permeability was ensured by a 0.1–0.3% detergent treatment. The ImmunoFISH method was developed specifically for studying meiosis in *Triticeae*, but should also be applicable to other grass and plant species.

## Introduction

Chromosome–chromosome interactions leading to homologous pairing at meiosis are accompanied by programmed changes in chromatin organisation and structure. Distinct stages of chromatin remodelling and movement with association of landmark chromosomal regions imprint the period of early meiosis and result in a distinctive nuclear polarisation (Colas et al., 2008; Heslop-Harrison and Schwarzacher, 2011; Sepsi et al., 2017). These chromatin dynamics are facilitated by meiosis-specific functional protein complexes, whose presence and localisation at particular chromosomal regions reflect the progression through meiosis (Schwarzacher, 2003; Cahoon and Hawley, 2016). To understand regulation and interdependence of these complex events, and to evaluate possible effects on plant fertility, the morphogenesis of specific proteins needs to be visualised in the context of chromatin organisation and movement.

Isolation of the *Arabidopsis thaliana* genes involved in synapsis, recombination and meiotic chromatin movement and the production of polyclonal antibodies against meiotic proteins led to a more accurate deciphering of the meiotic processes in higher plants (Osman et al., 2011; Pradillo et al., 2014; Mercier et al., 2015; Naranjo, 2015; Varas et al., 2015). Methods are available to track meiotic proteins in *Arabidopsis* (Chelysheva et al., 2010; Higgins et al., 2014) but optimal tissue preservation and achievement of high-quality immunosignal in grasses with their large chromosomes (30–100 times the size of *Arabidopsis*; Choulet et al., 2010; Heslop-Harrison and Schwarzacher, 2011) requires modifications of fixation, preparation and labelling techniques.

Analysis of meiosis of crop plants such as the *Triticeae*, where the crop is the seed, is particularly important as any meiotic aberrations directly affect fertility and grain yield and thus have major economic impacts. Cytogenetic techniques including conventional staining, chromosome banding and fluorescent *in situ* hybridisation (FISH) had been used earlier to study meiosis in bread wheat (*Triticum aestivum*, 2n = 6x = 42) and provided valuable knowledge on the pairing of the hexaploid chromosome set. However, a precise timing of chromosome behaviour at early meiosis could not be determined using these techniques alone. Cytological and anatomical criteria (chromatin morphology, anther length and position of the floret within the ear) are only indicative for differentiating stages of prophase I (Aragón-Alcaide et al., 1997; Schwarzacher, 1997; Maestra et al., 2002). On the other hand, whilst electron microscopy can detect lateral and central elements of the synaptonemal complex providing an accurate sequence of chromosome synapsis – thus accurately defining the meiotic sub-stages – the complex preparation reveals only the SC and it is not possible to identify the individual chromosomes (Holm, 1977; Jenkins, 1983; Wang and Holm, 1988).

ImmunoFISH combines FISH to label chromosomes and chromosome regions with immunohistochemistry and high-resolution microscopy, to study chromatin dynamics and specific meiotic proteins simultaneously, providing a precise timing of prophase I (Martín et al., 2017; Sepsi et al., 2017). The protocol presented here was developed for the analysis of meiotic cells of *Triticeae* and focused on the three-dimensional (3D) preservation of nuclei and long-term storage without the need for time-consuming embedding and tissue sectioning. Our protocol, is an all in one method and does not need relocating photographed nuclei with the first procedure [e.g., immunolabelling (IL)] after the second procedure (e.g., FISH) as reported by Martín et al. (2017). It addresses challenges presented when nuclear proteins, often difficult to preserve and requiring careful balanced conditions during IL, are to be detected at the same time as specific DNA sequences using *in situ* hybridisation that require different procedures to open up chromatin to allow penetration of the probe.

Here, we also give details of growing plants in growth cabinets to provide plentiful developmentally homogenous material with healthy ears undergoing meiosis. We followed the chromosome axis component ASY1 and the SC transverse filament protein ZYP1 with respect to active centromeres (CENH3 labelling) and telomeric repeat sequences (TRS-FISH) within male meiotic nuclei of hexaploid wheat (Armstrong et al., 2002; Houben and Schubert, 2003; Higgins et al., 2005) the procedure has been proven on barley and wheat–barley hybrid lines when studying alien centromere activity and chromosome elimination at meiosis. Our method will also be applicable to other *Triticeae* and grass species and to generic FISH protocols (Schwarzacher and Heslop-Harrison, 2000) that we developed in wheat relevant to all plant species with little modification.

The presented fixation and IL method can be used alone to detect proteins. However when combining IL with FISH (ImmunoFISH) it is challenging to balance fixation and permeabilisation treatments to preserve protein antigenicity and 3D nuclear architecture while ensuring cell permeability. We used a non-denaturing fixative (4% paraformaldehyde, PFA), which preserved chromatin and nuclear proteins but hindered penetration of antibodies and labelled FISH probes. Permeabilisation steps were subsequently adjusted by testing several treatments. One of the major limitations of non-denaturing fixation of plant tissues is that samples need to be subject to IL promptly (a few days after fixation) otherwise nuclear integrity would be compromised. Meiotic experiments in wheat as well as other plants, involve processing of a large amount of flowers, spikes, spikelets, and anthers within a short period of time (generally 1–2 weeks for a set of plants), quickly resulting in a large number of preparations and the need to proceed to IL limits the number of plants that can be analysed. We therefore focused on methods for the long-term storage of meiotic preparations prior to the ImmunoFISH procedure in order to facilitate work planning and to increase the number of plants processed.

## Materials and Methods

### Plant Materials

Seeds of spring wheat cv. “Chinese Spring” were sown in Jiffy pellet pots and grown at room temperature (RT) for 10 days or until they were 10 cm high. Plants were replanted in soil in 20 cm diameter pots and transferred to a greenhouse or growth cabinet and maintained at 20°C (±1°C) with 12 h light/day. Winter cultivars that require a period of cold treatment (vernalisation) in order to avoid a delay in ear initiation, were transferred to a vernalisation cabinet ahead of potting up and kept at 4°C with 12 h light/day for 6 weeks. Shorter days (8 h) with slightly higher temperatures (up to 8°C) can also be used.

Potted plants should be ideally grown in growth cabinets where environmental conditions (temperature, day length, humidity, light intensity) can be adjusted to their specific needs. Alternatively, plants can be grown in a glasshouse or outdoors during spring exploiting the natural increase in day length and temperature, but time interval to flowering will not be as predicable using these methods due to changing weather patterns. A standard cabinet program for wheat consists of 15°C day/10°C night (12 h/day) for an initial 4 weeks period. Day length and temperature should then be increased by 2 h and 2°C, respectively, every 3 weeks until the day-temperature reaches 19°C and the day length 16 h/day. For faster growing wheat varieties and other cereals these time intervals might need to be adjusted as reaching 19°C before meiosis is important for full fertility. Depending on the variety, wheat enters meiosis ∼8–10 weeks after vernalisation. Compared to wheat, barley genotypes reach meiosis earlier, while rye usually enters meiosis 1–2 weeks later.

### ImmunoFISH Experimental Procedures

For the following experimental procedures, different reagents and conditions were tested (**Supplementary Table 1**), but only those yielding the best results are given here.

### Collection of Meiotic Tissue and Fixation

**FIGURE 1:**
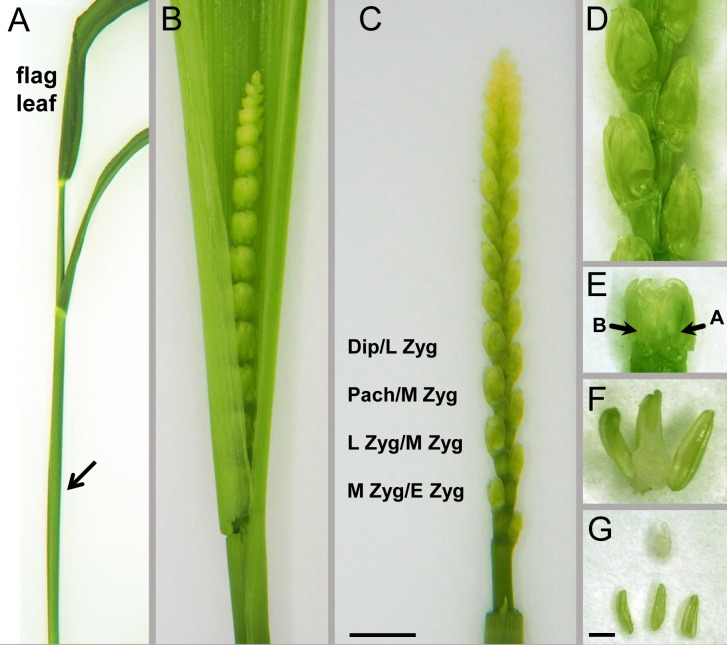
Dissection of anthers from the developing wheat spike. **(A)** An intact spike entering meiotic prophase I with the flag leave emerged and the ear enclosed within the sheaths of leaves (arrow). **(B)** The developing wheat ear within the manually opened leaf sheaths. **(C)** Dissected ear with a series of spikelets; each spikelet has a series of florets with decreasing developmental stages, example of stages as indicated. Dipl, diplotene; Pach, pachytene; Zyg, zygotene, L, late; M, middle; E, early. Bar = 1.5 cm in **(A)**, 1 cm in **(B)**, and 0.5 cm in **(C)**. **(D)** Alternating pattern of spikelets at the middle of the ear. **(E)** An individual spikelet with the two most developed florets termed as floret “A” and floret “B” (arrows). **(F,G)** Three anthers and the ovary dissected from an individual floret within a spikelet. Bar = 1 mm in **(D,E,G)**, 0.5 mm in **(F)**.

Meiotic division follows a circadian cycle, and meiocytes are arrested in early meiotic prophase during the night; in order to have stages around or in meiotic metaphase, spikes need to be collected in the morning ∼3 h after the light comes on. Developing ears within leaf sheaths can be felt by gently running the ear between the finger and thumb while squeezing slightly; their position in respect to internodes and the emergence of the flag leaf can be used for estimating ear development (**Figure 1**). Tillers estimated to enter meiosis were collected and placed in water to avoid desiccation. Spikes were then dissected from the leaf sheaths and transferred to a Petri dish containing a wet filter paper.

Spikes composed of a series of spikelets alternating on opposite sides of the rachis were numbered for reference starting at the bottom. Spikelets of hexaploid wheats carry at least three fertile florets (**Figure 1**), however, only the first two florets are used in most studies, since they are the best developed and most uniform. The most developed spikelets are located in the middle of the ear while spikelets towards the top and the base carry florets at successively earlier stages with the top and bottom spikelet, respectively, being the earliest. Anthers of the same floret carry meiocytes in the same developmental stage (Bennett, 1971). Starting with the spikelet positioned at the middle of the ear, one anther was excised from the first two florets (termed as floret A and B) and squashed in 45% (v/v) acetic acid to determine the approximate meiotic stage. Anther lengths were also carefully noted down for future reference. The two remaining anthers of each floret were placed into a 14-mm cell culture insert (8 μm membrane pore size, Thermo Fisher Scientific, Waltham, MA, United States, No. 140656) in a 12-well tissue culture plate containing ice-cold 4% PFA.

**FIGURE 2:**
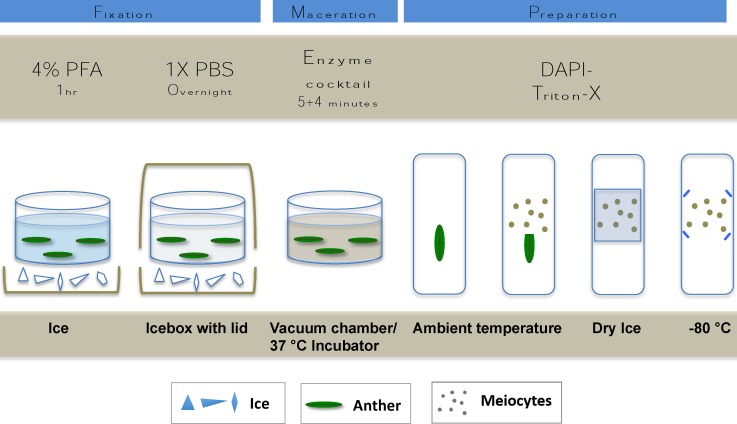
Cartoon showing the main steps of tissue fixation maceration and slide mounting of meiotic nuclei preparation.

Isotonic microfiltered PFA (Agar Scientific, Stansted, United Kingdom; No. R1026, 16%) was diluted to 4% in 1× PBS (phosphate buffered saline, 137 mM NaCl, 2.68 mM KCl, 10 mM Na_2_HPO_4_, 1.76 mM KH_2_PO_4_, pH 7.4). 4% PFA can alternatively be freshly prepared by dissolving PFA powder (Sigma-Aldrich, Darmstadt, Germany, No. P6148) in 1× PBS by heating to 75–80°C while stirring. A 48-μM NaOH/100 ml of fluid should be added (e.g., 6 μl of 8 M NaOH/100 ml) after which the mix will immediately clear. To avoid thermal decomposition, care should be taken not to overheat the solution. Sampling continued towards the bottom of the ear and anthers were consequently checked and fixed from the first two florets of each spikelet. In order to allow the penetration of PFA into the inner layers of the tissues, anthers were fixed for 1 h while kept on ice. Washing consisted of two times 5 min at RT in 1× PBS by keeping the anthers in the inserts and exchanging the fluids with a fine tip Pasteur pipette. Fixed and washed anthers were kept in a final change of fresh 1× PBS in the inserts of the plate that was covered with a lid and placed on ice into an icebox and left at 4°C overnight (**Figure 2**).

### Preparation of Cell Nuclei for ImmunoFISH

1× PBS washing buffer was removed carefully and replaced by 300 μl of enzyme solution prepared according to Houben and Schubert (2003). Final concentrations were as follows: 2.5% (w/v) pectinase (from *Aspergillus niger*, 1 U/mg protein Sigma-Aldrich, 17389) 2.5% (w/v) pectolyase (from *Aspergillus japonicus*, 0.3 U/mg protein, Sigma-Aldrich, P3026), 2.5% (w/v) cellulase (from *A. niger*, 0.3 U/mg protein, Sigma-Aldrich, C1184) and 1.5% (w/v) cytohelicase (extracted from *Helix pomatia*, Sigma-Aldrich, No. C8274); enzyme mixture can be stored frozen at -20°C for several months and can be used repeatedly. Five minutes of vacuum infiltration was used to facilitate enzyme penetration. Samples were subsequently incubated at 37°C for an additional 5 min. Anthers were kept on ice and subsequent sample preparation was made to judge the extent of digestion. Incubation with the enzyme mix could be extended for a further 5 min at 37°C if needed. The enzyme solution was then immediately taken off and replaced with ice-cold 1× PBS (two times for 5 min).

Preparations were made promptly after the enzyme treatment on Superfrost Plus Adhesion slides (Thermo Fisher Scientific). The anther tip was cut with a razor blade and using fine tungsten needles, pollen mother cell loculi were squeezed out into a drop of DAPI-Triton-X (2 μg/ml 4′,6-diamidino-2-phenylindole, Sigma-Aldrich, No. 10236276001; diluted from 100 μg/ml stock in water with 0.05% Triton-X in 1× PBS). A coverslip was carefully placed on the top of the cell mixture and tapped lightly to produce a single layer of meiotic cells then pressed gently between filter papers. The quality of preparations was examined by using an epifluorescent microscope and slides suitable for ImmunoFISH were snap-frozen on dry ice. Coverslips were removed by a razor blade and slides were either used immediately or quickly transferred to -80°C to avoid preparations drying out (**Figure 2**). Samples were stored for up to 12 months.

### FISH Probe Preparation

The universal plant TRS were amplified by PCR using the oligomer primers T1 (5′-TTTAGGG-3′)_5_ and T2 (5′-CCCTAAA-3′)_5_ (Schwarzacher and Heslop-Harrison, 1991). For preparing the barley genomic probe, total genomic DNA of barley was sheared and labelled by nick translation with biotin-14-dATP as described (Sepsi et al., 2008).

### Permeabilisation, Post-fixation, and *in situ* Hybridisation

Before *in situ* hybridisation, nuclei were permeabilised so that the FISH probes could access the chromatin threads. Several conditions and different detergents were tested (see section “Results”) before settling on the most successful as follows. Slide-mounted preparations were taken out from the ultra-freezer and immediately put into cold (4°C) 1× PBS buffer for 5 min. Slides were then transferred to 0.1% Triton-X and 0.3% CHAPS (3-[(3-cholamidopropyl)dimethylammonio]-1-propanesulfonate) hydrate (Sigma-Aldrich, No C3023) in 1× PBS and incubated at RT for 10–15 min and washed twice in 1× PBS. Subsequently, preparations were incubated in 10 mM HCl for 1 min at RT and 50 μl pepsin solution (100 μg/ml pepsin in 1 mM HCl, Sigma-Aldrich, No P6887 3,200-4,500 U/mg protein) was applied to each slide, covered with a parafilm cover slip and incubated at 37°C for 5 min. Slides were rinsed in distilled water followed by a quick post-fixation step in 4% PFA in 1× PBS (3 min at RT under a fume hood) and washed in 1× PBS.

Slides first underwent the FISH protocol (see Schwarzacher and Heslop-Harrison, 2000; Sepsi et al., 2008). Briefly, the probe mixture was prepared for each slide by adding 60 ng labelled TRS probe to 30 μl hybridisation mix consisting of 60% (v/v) deionised formamide (Sigma-Aldrich, No. F9037) and 10% (w/v) dextran sulphate (Sigma-Aldrich, No. 67578) in 2× SSC. The probe mixture was denatured at 85°C for 8 min 30 s in a PCR machine and immediately transferred to ice for at least 5 min. The ice-cold mixture was carefully pipetted on the surface of the preparations and covered with a 22 × 32 glass coverslip. The slides were denatured together with the probe mix at 75°C for 4 min on a PCR machine equipped with a stainless steel plate (see Sepsi et al., 2008). The slides were then transferred to a plastic box with a fitted lid containing moist tissue paper, and incubated at 37°C overnight. Post-hybridisation washes consisted washing slides four times in 1× PBS at 37°C 5 min each taking care that slides do not dry out between steps.

### Immunolabelling of Meiotic Proteins

Nuclei preparations were blocked immediately after the post-hybridisation washes by adding 50–100 μl of IS blocking buffer consisting of 1× TNB (0.1 M Tris–HCl, pH 7.5, 0.15 M NaCl, 0.5% Blocking Reagent, Roche, No. 11096176001) and 0.3 M Glycine (Sigma-Aldrich, G8898). A parafilm coverslip was applied and slides were replaced in the plastic box for 30–60 min at RT. Rabbit anti-CENH3 primary antibody was prepared as described by Sepsi et al. (2017). Guinea pig anti-ASY1 antibody was developed by an immunisation program using a recombinant protein including the Horma-domain of *A. thaliana* ASY1 protein while the ZYP1 antibody was produced against a recombinant protein matching the C-terminal region of the *A. thaliana* ZYP1B protein (Osman et al., 2018). Primary antibodies were diluted in a ratio of 1:300 in IS blocking buffer. A 50 μl of the diluted solution was added to each slide covered with a plastic coverslip and put back into the moist plastic box. Preparations were incubated for 3 h at 37°C then washed twice in 1× PBS at RT 5 min each again avoiding any drying out between steps.

In order to detect the localisation of the primary antibodies and that of the biotin FISH signal we used appropriate secondary antibodies depending on the animal that the primary antibodies were raised with and are as follows: goat anti-rabbit IgG, Alexa 594 or 488 (Invitrogen, CA, United States No. A-11072, A-11008), donkey anti-rat IgG, Alexa 488 or goat anti-rat IgG Alexa 594 (Invitrogen No. A-21208 and Abcam, Cambridge, United Kingdom, No. ab150160), goat anti-guinea-pig IgG, Alexa 647 (Abcam, No. ab150187) and streptavidin, Alexa 488 or Alexa 594 conjugates (Invitrogen, Nos. S11223, S11227). The working solution was prepared in IS blocking buffer with a final concentration of 1:300 for the secondary antibodies and a 4 μg/ml final concentration for the streptavidin. A 50 μl of the working solution was added to each slide, covered with a plastic coverslip and incubated for 30–45 min at 37°C. After washing the slides twice in 1× PBS, 12 μl of Vectashield Antifade Mounting Medium with DAPI (Vector Laboratories, Burlingame, CA, United States, No. H-1200) was applied per slide and covered with a thin 22 or 24 × 32 No. 0 glass coverslip.

### Image Collection

Confocal microscopy was carried out using a Leica TCS SP8 confocal laser-scanning microscope (Leica Microsystems GmbH, Wetzlar, Germany). Series of confocal images (“*z* stacks”) with a lateral (*x* and *y*) resolution of 45 nm and an axial (*z*) resolution of 200 nm were acquired by a HC PL APO CS2 63×/1.40 oil immersion objective (Leica Microsystems GmbH). Size of confocal aperture was set to 1.35 Airy Unit (128.9 μm). Image acquisition was carried out by bidirectional scanning along the *x*-axis, and images were averaged from three distinct image frames in order to reduce image noise. Image stack deconvolution was performed using a Huygens Essential software v17.10 (Scientific Volume Imaging, Hilversum, Netherlands). 3D reconstructions were obtained using a Leica Application Suite Advanced Fluorescence software v3.1.5.1638 (Leica Microsystems GmbH).

ImmunoFISH images of wheat–barley introgression lines were captured by widefield microscopy on a Nikon Eclipse 80i epifluorescent microscope equipped with DS-QiMc monochromatic camera (Nikon, Tokyo, Japan). Series of grey-scale images for each colour were captured along the *Z*-axis, taking 15–20 stacks per colour. Single channel images were pseudocoloured and merged in NIS elements (Nikon). All-in-focus images were created using the extended depth of focus (EDF) module.

## Results

**FIGURE 3:**
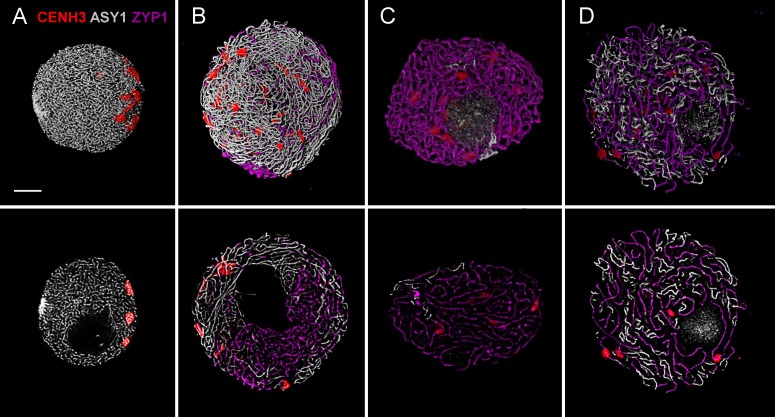
Three-colour immunolabelling on meiotic prophase I (leptotene to diplotene) nuclei of wheat. CENH3 centromeric protein is visualised by a rabbit anti-CENH3 antibody and is shown in red, SC axial element protein ASY1 is visualised by a guinea pig anti-ASY1 antibody and is shown in grey and SC central element protein is visualised by a rat anti-ZYP1 antibody and is pseudo-coloured in purple. Three-dimensionally reconstructed confocal image stacks are shown in the top row while a single *z* stack of the image is presented in the bottom row. Images show substages of meiotic prophase I: **(A)** leptotene, **(B)** mid-zygotene, **(C)** pachytene, **(D)** diplotene. Bar = 5 μm.

**FIGURE 4:**
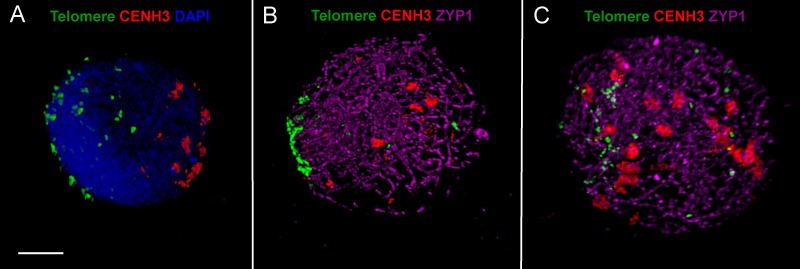
ImmunoFISH showing progression of synapsis and simultaneous centromere–telomere dynamics through three successive stages of meiotic prophase I in wheat. **(A)** Early leptotene, **(B)** early-mid zygotene, **(C)** late zygotene. Active centromeres and the SC central element protein are visualised by immunolabelling with anti-CENH3 (rabbit, red) and anti-ZYP1 (rat, purple) antibodies while telomeres are detected by TRS-FISH (green). Bar = 5 μm.

**FIGURE 5:**
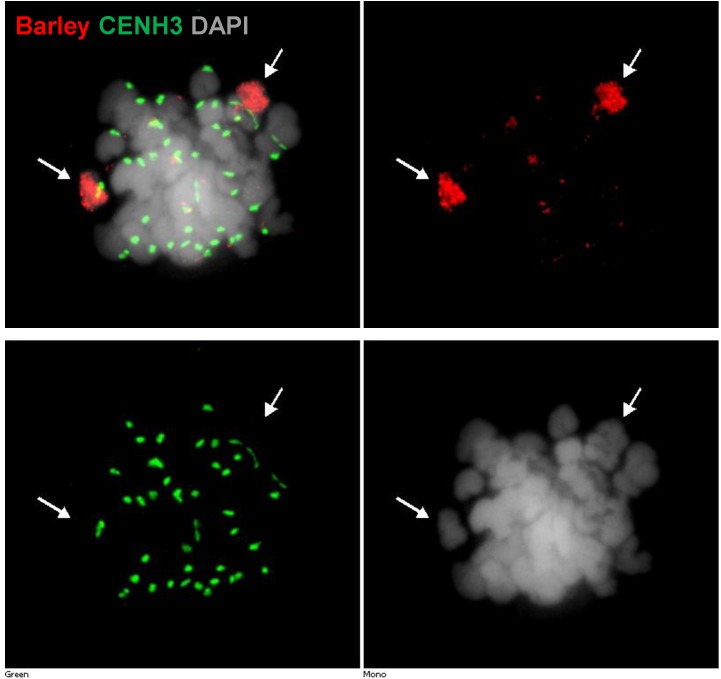
Detection of barley chromatin and CENH3 centromeric histone protein within the metaphase chromosomes of a wheat–barley addition line (2n = 6x = 44) carrying a pair of 3H chromosome and the complete chromosome complement of wheat. Barley chromatin is shown in red (arrows) and CENH3 is visualised in green colour labelled by a rabbit-anti CENH3 antibody. Chromosomes are counterstained by DAPI (grey signal). Bar = 5 μm.

By using our method, chromatin dynamics could be mapped in high resolution within the meiotic nuclei of bread wheat. We were able to visualise nuclear structures (telomeres, centromeres, SC axial and central element related proteins) simultaneously with the bulk chromatin (DAPI contrast staining) and followed changes in organisation and structure throughout meiotic prophase I (**Figures 3**–**5**). Proteins and landmark chromosome regions can be detected together at 2D or 3D by conventional epifluorescence microscopy, however, resolution, especially along the *z*-axis will be low. As nuclei were only squashed lightly during preparation, this allowed us to observe meiotic nuclei in 3D by using optical sectioning reaching a resolution of 45 nm in the *x*- and *y*-directions, and 150–200 nm in the *z*-direction. By sampling nuclei of different developmental stages from anthers of florets at defined positions within the ears (**Figure 1**), a sequence of meiotic chromatin events could be developed.

### Preservation of Nuclei and Proteins

Good spatial visualisation of cellular structures, such as we were able to observe (**Figures 3**, **4**), assumes meiotic cells to be adequately preserved, stored and handled during the staining procedures. Cells fixed with PFA have a superior cellular architecture and proteins are preserved in their 3D relationships within the nucleus (Kiernan, 2000). However, in order to access nuclear structures during IL and FISH, only a very short PFA fixation (1–2 h) at 4°C with immediate transfer to buffer can be undertaken (**Supplementary Table 1**) and therefore keeping those fixed plant tissues at RT or 4°C would shortly lead to sample degradation and quality loss. Similarly, keeping ready prepared slides at RT also showed sample degradation.

In order to store slides and plan experiments with large number of samples, we investigated the effect of deep-freezing on the preservation of preparations. Storage of slides at -20°C saw a rapid decrease in quality, however, snap freezing followed by storage at -80°C preserved the 3D structure for up to 12 months (**Supplementary Table 1**) and the quality of the stored slides was comparable to those of the fresh samples. For a good structural preservation, it is important to avoid preparations drying out throughout all steps of the procedure.

### Permeabilisation of Tissue

This fixation method can be used to detect proteins using IL. However, when combined with FISH one needs to balance fixation and permeabilisation treatments in order to preserve protein antigenicity and 3D nuclear architecture, whilst ensuring cell permeability. We used a non-denaturing fixative (4% PFA), which preserved chromatin and nuclear proteins but hindered penetration of antibodies and labelled FISH probes. We tested several concentrations and combinations of permeabilisation agents (cold methanol, Proteinase-K, RNase, Tween-20, Triton-X-100, CHAPS-hydrate, repeated freeze-thaw cycles; see **Supplementary Table 1**) for various times prior to ImmunoFISH to achieve an optimal accessibility of DNA and proteins whilst maintaining nuclear structure. The best results were obtained with a low concentration of Triton-X-100 and CHAPS hydrate-solution (0.1 and 0.3%, respectively, in 1× PBS) followed by a short pepsin digestion and a quick PFA post-fixation step applied before FISH (**Supplementary Table 1**). NICK-Translation *in situ* probe labelling was chosen in order to control probe-length (the longer the Nick-translation procedure the shorter the probe) and thus further facilitate penetration, but assume that short directly labelled oligonucleotides or those labelled with random priming will also be suitable.

### Precise Protein Immunolocalisation During Meiosis

Following adequate fixation and permeabilisation treatments we obtained well-preserved meiotic nuclei and were able to label the following nuclear structures with IL: CENH3 protein (showing active centromeres) together with SC axial and central element proteins ASY and ZYP1. Subsequently, in separate experiments we performed ImmunoFISH to show telomeres and centromeres concurrently with SC central elements (ZYP1): telomeres were visualised by FISH (TRS-FISH) while centromeres and the ZYP1 proteins were stained by IL. The bulk chromatin was labelled by DAPI in both cases. This enabled us to correlate centromere–telomere dynamics and that of the bulk chromatin to the development of the synaptonemal complex (Sepsi et al., 2017; **Figures 3**, **4**).

Samples were scanned by confocal laser scanning microscopy (CLSM) and 2D images were collected every 200 nm in depth (*Z*-direction) from each channel resulting in *z* stack series covering the whole 3D volume of the nucleus (**Figures 7A–D**, **8A,B,F,G**). Consequently, merged 2D images of the four channels collected from the same focal plane represented localisation of the nuclear structures within the *x*- and *y*-axis (**Figures 3** bottom row, **6**, **7E**, **8C–E**) while 3D reconstruction of the whole *Z*-series allowed modelling of spatial relationships within the nucleus (**Figures 3** top row, **7A–D**, **8**).

**FIGURE 6:**
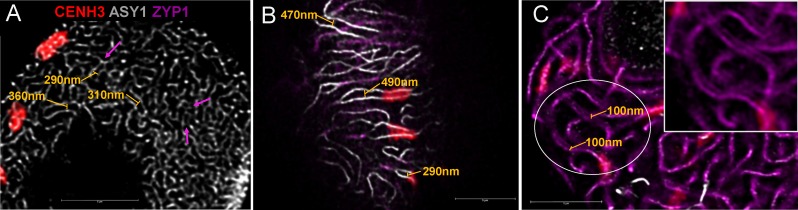
Axis distances during early meiotic prophase I in hexaploid wheat. **(A)** Leptotene and **(B)** zygotene images are single stack enlargements from samples presented in **Figures 3A,B**. Panel **(C)** is from a pachytene nucleus. Unsynapsed chromosome axes (later lateral elements) are shown as white threads (guinea pig anti-ASY1 antibody, detected by Alexa 647), the central element protein of the synaptonemal complex (rat anti-ZYP1 antibody, is pseudocoloured in purple and active centromeres (rabbit anti-CENH3 antibody) are displayed in red. Axis distances are indicated by yellow bars on **(A)** and **(B)** and purple arrows highlight closely aligned, coalescing chromosome axes. Pachytene double structures **(C)** seen with the anti-ZYP1 antibody are highlighted by yellow bars; a region of interest is enlarged and inserted to the upper right of the figure). Bar = 5 μm.

As a single ear of a cereal plant contains a sequence of meiotic stages, sampling spikelets/anthers from the middle towards the bottom or top of the ear revealed dynamics of nuclear structures during meiotic prophase I. Centromere- and telomere associations, their position with respect to the nuclear envelope and the state of SC formation are reliable indicators of meiotic progression and can be referred to as meiotic markers showing sub-stages of prophase I (Sepsi et al., 2017). Leptotene nuclei, defined by the formation of major polarised centromere associations and the linearisation of axial elements along chromosomes, showed in addition to our previous results (Sepsi et al., 2017) a notable nuclear compaction (**Figure 3A**). Chromosome axes were compressed against each-other frequently forming parallel alignments of less than 300 nm while some regions showed two separate chromosome axes coming near each other and coalescing through protein bridges (**Figure 6A**). During zygotene, when synapsis elongates in two stages (first from the telomeres and second from the chromosome arms), nuclei became enlarged and parallel alignments between the unsynapsed axes measured 300–500 nm, exceeding axis distances observed at leptotene (**Figures 3B**, **6B**). At pachytene, bivalents show perfect synapsis and when labelled with anti-ZYP1 antibody juxtaposed axes were discerned as double-structures 100 nm apart (**Figure 6C**). Asymmetric arrangement typical of early prophase nuclei resolved at late zygotene when the telomere bouquet dispersed (Sepsi et al., 2017; **Figure 4**) and during desynapsis in diplotene landmark chromosomal regions showed random distribution (**Figures 3C,D**).

We also used ImmunoFISH detected by widefield fluorescence microscopy to follow centromere activity of alien chromosomes in wheat–barley introgression lines (**Figure 5**). Labelled total genomic DNA of barley and CENH3 antibody showed active centromeres demonstrating that barley centromeres successfully load wheat CENH3, (**Figure 5**).

We analysed early meiosis in diploid barley cv. Golden Promise (2n = 2x = 14) and in a synthetic tetraploid barley (2n = 4x = 28) and showed association of telomeres in small groups scattered within the nucleus during the period of axis extension in early leptotene which was followed by telomere bouquet formation (**Figures 8C–E**). Leptotene was marked by a polarised chromatin organisation whereby telomeres were gathered close to the nuclear envelope and centromeres were associated in small groups at the periphery opposite to the telomere bouquet (**Figures 8A,B,F,G**).

**FIGURE 7:**
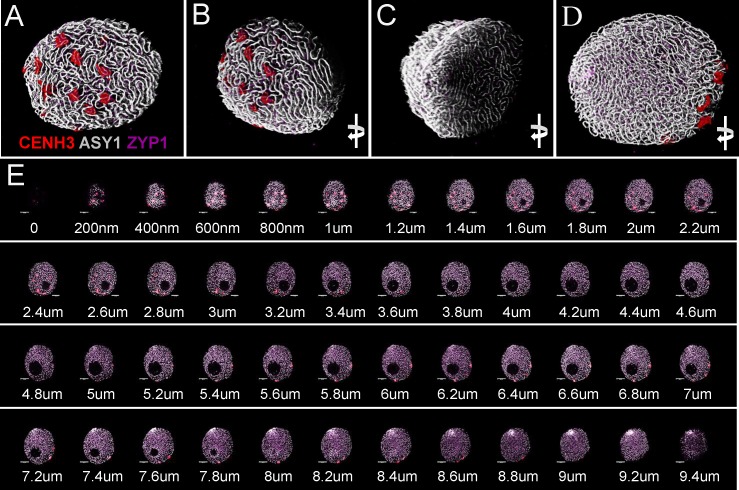
Three-dimensional rendering of 2D confocal images collected from sequential focal planes of an early zygotene nucleus. Different channels represent signals from anti-CENH3 (red), anti-ASY1 (white), and anti-ZYP1 (purple) antibodies. The 3D image is turned to be observable from different angles. **(A)** Centromeric pole, **(B)** side view of the centromeric pole, **(C)** telomeric pole with the telomere bouquet, **(D)** projection turned 180° compared to the centromeric pole. **(E)** Optical sections collected by confocal laser scanning microscopy showing sequential focal planes of **(A–D)**. Δ*Z* = 200 nm. Bar = 5 μm.

**FIGURE 8:**
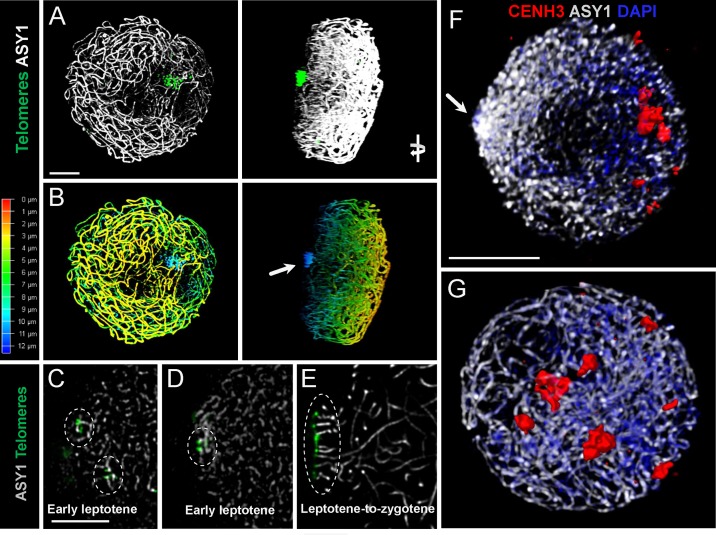
Spatial relationships of telomeres, centromeres, and chromosome axes in early meiosis of a diploid and a synthetic tetraploid barley (2n = 4x = 28, originating from a cross between two autotetraploid *Hordeum vulgare* cultivars: Morex × Golden Promise). Axial elements are visualised by an anti-ASY1 antibody (white signal), centromeres are stained with an anti-CENH3 antibody and shown in red, while telomeres are detected by FISH (green signal in **A,C–E**). Chromatin is contrast stained with DAPI (blue) on **(F,G)**. **(A)** Snapshot of a 3D rendered early zygotene nucleus with continuous chromosome axes and fully formed telomere bouquet in the tetraploid barley. The nucleus is turned to show the side view. **(B)** Conversion of the 3D rendered stack into a 2D image by *z*-depth-coding, whereby consecutive *z*-slices are represented by a specific colour (colour coding on the left). Note the extreme peripheral localisation of the telomeres perceived in deep blue (= 12 μm in depth, arrow). **(C,D)** Single stack images showing early telomere behaviour in tetraploid barley with small telomere groups (encircled) scattered within the nucleus in early leptotene while chromosome axes are yet uncontinuous (white signal). **(E)** At the leptotene-to-zygotene transition telomeres become gathered within a small peripheral area of the nucleus to form the bouquet while subtelomeric chromosome axes align in anticipation of pairing (white parallel threads, encircled). **(F)** Uncontinuous chromosome axes within a polarised leptotene nucleus and small centromere groups located close to the nuclear periphery while the telomere bouquet is formed at the opposite pole (arrow). **(G)** Axial elements are linear and centromeres become released from the nuclear periphery during zygotene.

## Discussion

Here we present a simple and reliable protocol for the IL of meiotic proteins within higher plants, combining IL with FISH, offering the advantage of simultaneously tracking multiple proteins and specific DNA sequences. Previous methods providing visualisation of high quality 3D nuclear structures in plants required laborious tissue fixation, embedding and sectioning (Holm, 1977; Murphy and Bass, 2012) and have limited possibilities for the identification of specific proteins and DNA sequences. Sectioning of the material after short fixation using a vibratome can take several days despite the need for processing plant material promptly following a brief fixation (Aragon-Alcaide et al., 1998; Prieto et al., 2007) risking the degradation of material. Our technique produces a single layer of well-preserved, cytoplasm-free nuclei embedded in antifade, an ideal situation for high resolution imaging with CLSM. Good resolution using CLSM on thick sections is only possible within the upper 15–20 μm of the preparations because of the light scattering effect of the cytoplasm and the cell walls, reducing the efficiency of both fluorochrome excitation and emitted signal detection. Although point spread function of the imaging system can be corrected using a deconvolution software, signal-reducing effect of the thick tissue surrounding the nuclei results in lower quality compared to the single-layer preparations. Our method does not depend upon specialised sectioning equipment and can be completed within two working days following slide preparation. Moreover, meiotic preparations can be stored for several months in an ultra-freezer without any effect on nuclear morphology, allowing a better project planning and processing of a significantly higher number of plants.

ImmunoFISH was used earlier to follow chromatin dynamism during SC formation in hexaploid wheat and revealed that major centromere associations followed by the formation of the telomere bouquet create a highly polarised nuclear environment that immediately precedes synapsis initiation from the telomeres. We showed that centromere dynamics (notably depolarisation and scattering of centromere clusters) correlates with a second wave of SC elongation from multiple nucleation sites within the euchromatic chromosome arms (Sepsi et al., 2017).

In the present work, we were able to increase the resolution of our analysis, and to measure axis distances through meiotic prophase I and showed alignments of 200–300 nm at leptotene. In contrast, parallel configurations of unsynapsed regions at mid-zygotene spanned 300–500 nm indicating that the tight chromatin arrangement observed in early meiosis is relaxed upon SC initiation. Presynaptic alignments of 400 nm typical of leptotene nuclei are known to be mediated by recombination initiation through the interactions of homologous duplexes (Henderson and Keeney, 2005). A detailed investigation in the filamentous fungus *Sordaria macrospora* (Storlazzi et al., 2010) indicated that homologous juxtapositions at leptotene are promoted by proteins of the recombination machinery (Mer3, Msh4, and Mlh1) with Msh4 identified as the first factor specifically determining presynaptic inter-axis distances. Thus, specific recombination complex proteins support homologous pairing well before their action in the recombination process. Double strand break (DSB)-mediated homologue interactions also define spatial patterns of SC nucleation sites, a subset of which carry embedded crossover interactions (Zhang et al., 2014). Larger axis distances as we showed at mid-zygotene, when a great proportion of the chromosome arms were synapsed, are most likely indicative of synapsis elongating from earlier nucleation sites rather than new SC initiation sites.

Protein morphogenesis can reflect initiation and progression of recombination, strategy of synapsis construction in wild and mutant backgrounds and thus is a major indicator of the fate of meiosis. Alteration in chromatin organisation and dynamics are essential to generate chromosome–chromosome interactions during the critical period of homologue recognition and pairing, so the timing and fidelity of synapsis and recombination relies on these processes (Lambing et al., 2015; Székvölgyi et al., 2015). Meiosis is influenced by environmental factors so it is increasingly relevant to reveal meiotic progression under stress conditions in crops and highlight the variable exposures influencing recombination, fertility and ultimately yield (Jackson et al., 2015; Phillips et al., 2015; Modliszewski and Copenhaver, 2017). To understand the control and interdependence of key meiotic events in higher plants it is critical to simultaneously study DNA processes and chromatin organisation. Our method has allowed us to analyse chromatin dynamics at meiosis with high resolution and will be applicable to plant species in general to gain insight into this critical stage of plant reproduction.

## Data Availability

All datasets generated for this study are included in the manuscript.

## Author Contributions

TS, JH-H, and AS conceived and designed the project. AS made the chromosome preparations and performed the cytological experiments. AF and KJ designed the settings and carried out the confocal microscopy analysis. AS and TS wrote the paper. All authors contributed to the manuscript revision, and read and approved the submitted version.

## Conflict of Interest Statement

The authors declare that the research was conducted in the absence of any commercial or financial relationships that could be construed as a potential conflict of interest.

## References

[B1] Aragon-Alcaide L. Beven A. Moore G. Shaw P. (1998). The use of vibratome sections of cereal spikelets to study anther development and meiosis. *Plant J.* 14 503–508. 10.1046/j.1365-313X.1998.00148.x

[B2] Aragón-Alcaide L. Reader S. Beven A. Shaw P. Miller T. Moore G. (1997). Association of homologous chromosomes during floral development. *Curr. Biol.* 7 905–908. 10.1016/S0960-9822(06)00383-6 9382806

[B3] Armstrong S. J. Caryl A. P. Jones G. H. Franklin F. C. (2002). Asy1 a protein required for meiotic chromosome synapsis, localizes to axis-associated chromatin in Arabidopsis and Brassica. *J. Cell Sci.* 115 3645–3655. 10.1242/jcs.00048 12186950

[B4] Bennett M. D. (1971). The duration of meiosis. *Proc. R. Soc. Lond.* 178 277–299. 10.1098/rspb.1971.0066

[B5] Cahoon C. K. Hawley R. S. (2016). Regulating the construction and demolition of the synaptonemal complex. *Nat. Struct. Mol. Biol.* 23 369–377. 10.1038/nsmb.3208 27142324

[B6] Chelysheva L. Grandont L. Vrielynck N. Le Guin S. Mercier R. Grelon M. (2010). An easy protocol for studying chromatin and recombination protein dynamics during *Arabidopsisthaliana* meiosis: immunodetection of cohesins, histones and MLH1. *Cytogenet. Genome Res.* 129 143–153. 10.1159/000314096 20628250

[B7] Choulet F. Wicker T. Rustenholz C. Paux E. Salse J. Leroy P. (2010). Megabase level sequencing reveals contrasted organization and evolution patterns of the wheat gene and transposable element spaces. *Plant Cell* 22 1686–1701. 10.1105/tpc.110.074187 20581307 PMC2910976

[B8] Colas I. Shaw P. Prieto P. Wanous M. Spielmeyer W. Mago R. (2008). Effective chromosome pairing requires chromatin remodeling at the onset of meiosis. *Proc. Natl. Acad. Sci. U.S.A.* 105 6075–6080. 10.1073/pnas.0801521105 18417451 PMC2329686

[B9] Henderson K. A. Keeney S. (2005). Synaptonemal complex formation: where does it start? *Bioessays* 27 995–998. 10.1002/bies.20310 16163735

[B10] Heslop-Harrison J. S. (2000). comparative genome organization in plants: from sequence and markers to chromatin and chromosomes. *Plant Cell Online* 12 617–636. 10.1105/tpc.12.5.617 PMC139916 10810139

[B11] Heslop-Harrison J. S. Schwarzacher T. (2011). Organisation of the plant genome in chromosomes. *Plant J.* 66 18–33. 10.1111/j.1365-313X.2011.04544.x 21443620

[B12] Higgins J. D. Sanchez-Moran E. Armstrong S. J. Jones G. H. Franklin F. C. (2005). The Arabidopsis synaptonemal complex protein ZYP1 is required for chromosome synapsis and normal fidelity of crossing over. *Genes Dev.* 19 2488–2500. 10.1101/gad.354705 16230536 PMC1257403

[B13] Higgins J. D. Wright K. M. Bomblies K. Franklin F. C. (2014). Cytological techniques to analyze meiosis in *Arabidopsis arenosa* for investigating adaptation to polyploidy. *Front. Plant Sci.* 4:546. 10.3389/fpls.2013.00546 24427164 PMC3879461

[B14] Holm P. B. (1977). Three-dimensional reconstruction of chromosome pairing during the zygotene stage of meiosis in *Lilium longiflorum* (thunb.). *Carlsberg Res. Commun.* 42 103–151. 10.1007/BF02906489

[B15] Houben A. Schubert I. (2003). DNA and proteins of plant centromeres. *Curr. Opin. Plant Biol.* 6 554–560. 10.1016/j.pbi.2003.09.007 14611953

[B16] Jackson S. Nielsen D. M. Singh N. D. (2015). Increased exposure to acute thermal stress is associated with a non-linear increase in recombination frequency and an independent linear decrease in fitness in Drosophila. *BMC Evol. Biol.* 15:175. 10.1186/s12862-015-0452-8 26310872 PMC4551699

[B17] Jenkins G. (1983). Chromosome pairing in triticum aestivum cv. *Chin. Spring. Carlsberg Res. Commun.* 48 255–283. 10.1007/BF02907772

[B18] Kiernan J. A. (2000). Formaldehyde, formalin, paraformaldehyde and glutaraldehyde: what they are and what they do. *Micros. Today* 8 8–13. 10.1017/S1551929500057060

[B19] Lambing C. Osman K. Nuntasoontorn K. West A. Higgins J. D. Copenhaver G. P. (2015). Arabidopsis PCH2 mediates meiotic chromosome remodeling and maturation of crossovers. *PLoS Genet.* 11:e1005372. 10.1371/journal.pgen.1005372 26182244 PMC4504720

[B20] Maestra B. Hans de Jong J. Shepherd K. Naranjo T. (2002). Chromosome arrangement and behaviour of two rye homologous telosomes at the onset of meiosis in disomic wheat-5RL addition lines with and without the Ph1 locus. *Chromosome Res.* 10 655–667. 10.1023/A:1021564327226 12575794

[B21] Martín A. C. Rey M.-D. Shaw P. Moore G. (2017). Dual effect of the wheat Ph1 locus on chromosome synapsis and crossover. *Chromosoma* 126 669–680. 10.1007/s00412-017-0630-0 28365783 PMC5688220

[B22] Mercier R. Mézard C. Jenczewski E. Macaisne N. Grelon M. (2015). The molecular biology of meiosis in plants. *Annu. Rev. Plant Biol.* 66 297–327. 10.1146/annurev-arplant-050213-035923 25494464

[B23] Modliszewski J. L. Copenhaver G. P. (2017). Meiotic recombination gets stressed out: CO frequency is plastic under pressure. *Curr. Opin. Plant Biol.* 36 95–102. 10.1016/j.pbi.2016.11.019 28258986

[B24] Murphy S. P. Bass H. W. (2012). The maize (Zea mays) desynaptic (dy) mutation defines a pathway for meiotic chromosome segregation, linking nuclear morphology, telomere distribution and synapsis. *J. Cell Sci.* 125 3681–3690. 10.1242/jcs.108290 22553213

[B25] Naranjo T. (2015). Contribution of structural chromosome mutants to the study of meiosis in plants. *Cytogenet. Genome Res.* 147 55–69. 10.1159/000442219 26658116

[B26] Osman K. Higgins J. D. Sanchez-Moran E. Armstrong S. J. Franklin F. C. (2011). Pathways to meiotic recombination in *Arabidopsis thaliana*. *New Phytol.* 190 523–544. 10.1111/j.1469-8137.2011.03665.x 21366595

[B27] Osman K. Yang J. Roitinger E. Lambing C. Heckmann S. Howell E. (2018). Affinity proteomics reveals extensive phosphorylation of the Brassica chromosome axis protein ASY1 and a network of associated proteins at prophase I of meiosis. *Plant J.* 93 17–33. 10.1111/tpj.13752 29078019 PMC5767750

[B28] Phillips D. Jenkins G. Macaulay M. Nibau C. Wnetrzak J. Fallding D. (2015). The effect of temperature on the male and female recombination landscape of barley. *New Phytol.* 208 421–429. 10.1111/nph.13548 26255865

[B29] Pradillo M. Varas J. Oliver C. Santos J. L. (2014). On the role of AtDMC1 AtRAD51 and its paralogs during Arabidopsis meiosis. *Front. Plant Sci.* 5:23. 10.3389/Fpls.2014.00023 24596572 PMC3925842

[B30] Prieto P. Moore G. Shaw P. (2007). Fluorescence in situ hybridization on vibratome sections of plant tissues. *Nat. Protoc.* 2 1831–1838. 10.1038/nprot.2007.265 17641652

[B31] Schwarzacher T. (1997). Three stages of meiotic homologous chromosome pairing in wheat: cognition, alignment and synapsis. *Sex. Plant Reprod.* 10 324–331. 10.1007/s004970050106

[B32] Schwarzacher T. (2003). Meiosis, recombination and chromosomes: a review of gene isolation and fluorescent in situ hybridization data in plants. *J. Exp. Bot.* 54 11–23. 10.1093/jxb/ 12456751

[B33] Schwarzacher T. Heslop-Harrison J. S. (1991). *In situ* hybridization to plant telomeres using synthetic oligomers. *Genome* 34 317–323. 10.1139/g91-052

[B34] Schwarzacher T. Heslop-Harrison J. S. (2000). *Practical in Situ Hybridization.* Oxford: BIOS, 90–124.

[B35] Sepsi A. Higgins J. D. Heslop-Harrison J. S. Schwarzacher T. (2017). CENH3 morphogenesis reveals dynamic centromere associations during synaptonemal complex formation and the progression through male meiosis in hexaploid wheat. *Plant J.* 89 235–249. 10.1111/tpj.13379 27624968

[B36] Sepsi A. Molnár I. Szalay D. Molnár-Láng M. (2008). Characterization of a leaf rust-resistant wheat-*Thinopyrum ponticum* partial amphiploid BE-1 using sequential multicolor GISH and FISH. *Theor. Appl. Genet.* 116 825–834. 10.1007/s00122-008-0716-4 18224300

[B37] Storlazzi A. Gargano S. Ruprich-Robert G. Falque M. David M. Kleckner N. (2010). Recombination proteins mediate meiotic spatial chromosome organization and pairing. *Cell* 141 94–106. 10.1016/j.cell.2010.02.041 20371348 PMC2851631

[B38] Székvölgyi L. Ohta K. Nicolas A. (2015). Initiation of meiotic homologous recombination: flexibility, impact of histone modifications, and chromatin remodeling. *Cold Spring Harb. Perspect. Biol.* 7:a016527. 10.1101/cshperspect.a016527 25934010 PMC4448624

[B39] Varas J. Graumann K. Osman K. Pradillo M. Evans D. E. Santos J. L. (2015). Absence of SUN1 and SUN2 proteins in *Arabidopsis thaliana* leads to a delay in meiotic progression and defects in synapsis and recombination. *Plant J.* 81 329–346. 10.1111/tpj.12730 25412930

[B40] Wang X. Holm P. B. (1988). Chromosome pairing and synaptonemal complex formation in wheat-rye hybrids. *Carlsberg Res. Commun.* 53 167–190. 10.1007/BF02907178

[B41] Zhang L. Espagne E. de Muyt A. Zickler D. Kleckner N. E. (2014). Interference-mediated synaptonemal complex formation with embedded crossover designation. *Proc. Natl. Acad. Sci. U.S.A.* 111 E5059–E5068. 10.1073/pnas.1416411111 25380597 PMC4250137

