# Supplementary material for: ImmunoFISH: Simultaneous Visualisation of Proteins and DNA Sequences Gives Insight Into Meiotic Processes in Nuclei of Grasses
Source: Front Plant Sci. 2018 Aug 14;9:1193. doi: 10.3389/fpls.2018.01193 (PMC6102387; doi:10.3389/fpls.2018.01193)
Supplement: Supplementary file 1 [file Data_Sheet_1.PDF]

Supplementary Material

ImmunoFISH: simultaneous visualisation of proteins and DNA sequences gives insight into meiotic processes in nuclei of grasses

A. Sepsi[1*], A. Fábián[1], K. Jäger[1] J. S. Heslop-Harrison[2], T. Schwarzacher[2]

Correspondence*

Adel Sepsi

sepsi.adel@agrar.mta.hu

Supplementary Table 1: Conditions and reagents tested to develop the presented protocols and our recommendations. Washes are not repeated in our recommendations when only one conditions was tested. Details of reagents and buffers are given in Material and Methods.

	Treatment					
	Main reagents	Additional reagents or conditions	Time and temperature[1)]	Wash	Storage	Recommended conditions
Fixation	4% PFA[2)] in 1x PBS	0.5% Tween<br>5-10 mins vacuum infiltration	0.5-12h<br>on ice, 4°C or RT	1x PBS twice for 5 mins each on ice or RT	On ice or 4°C<br>1-2 days	Fix for 1h on ice<br>No additional reagents<br>Wash twice in 1x PBS at RT<br>1 day storage on ice
Maceration	Enzyme cocktail[3]):<br>2.5% pectinase<br>2.5% pectolyase<br>2.5% cellulase<br>2.5-1.5% cytohelicase<br>in 1x PBS	5-10 mins vacuum infiltration	5-30 mins<br>RT or 37°C	1x PBS twice for 5 mins each on ice or RT	0-1 day	Enzyme cocktail with 1.5% cytohelicase<br>5 mins vacuum at RT followed by 5 mins 37°C<br>Wash twice in 1x PBS on ice<br>No storage
Chromo-some preparation	1x PBS containing 2µg/ml DAPI	0.05%-0.25% Triton-X			4°C, -20°C, or -80°C<br>1 day to 12 months	0.05% Triton-X in 1x PBS/DAPI<br>Storage at -80°C<br>6-12 months
Blocking procedures	TNB buffer	0.3M glycine	0.5-1h<br>RT	1x PBS twice for 5 mins each on ice or RT		TNB/0.3M glycine<br>30-60 mins<br>RT
Permeabilization	0.1% Triton-X and 0.3% CHAPS in 1x PBS	50µg/ml proteinase-K or 100-200 µg/ml pepsin<br>Ice cold 100% methanol	5-20 mins<br>RT or 37°C	1x PBS or distilled water twice<br>1 secs - 5 mins<br>RT		0.1% Triton-X / 0.3% CHAPS, 10-15 mins followed by 100µg/ml pepsin, 5 mins at 37°C
Post-fixation	4% PFA		3-10 mins<br>RT	1x PBS twice for 5 mins each at RT		4% PFA<br>3 mins<br>RT

1) RT (room temperature): 18 - 22 °C; 2) PFA: paraformaldehyde

3) Enzyme sources are given in Materials and Methods; if other sources are used, concentrations might need adjusting
